# Beyond Karl Fischer titration: a monolithic quantum cascade sensor for monitoring residual water concentration in solvents†

**DOI:** 10.1039/d2lc00724j

**Published:** 2023-02-13

**Authors:** Florian Pilat, Benedikt Schwarz, Bettina Baumgartner, Daniela Ristanić, Hermann Detz, Aaron M. Andrews, Bernhard Lendl, Gottfried Strasser, Borislav Hinkov

**Affiliations:** a Institute of Solid State Electronics and Center for Micro- and Nanostructures, TU Wien 1040 Vienna Austria florian.pilat@tuwien.ac.at borislav.hinkov@tuwien.ac.at; b Institute of Chemical Technologies and Analytics, TU Wien 1060 Vienna Austria; c Central European Institute of Technology (CEITEC), Brno University of Technology 621 00 Brno Czech Republic

## Abstract

Quality control of liquids is an important part of analytical chemistry. The gold standard for measuring residual water in organic solvents and pharmaceutical applications is Karl Fischer titration. It has a high sensitivity, selectivity and accuracy. The downsides are a time-consuming offline analysis, together with the need for toxic reagents producing waste, and it suffers from poor inter-laboratory reproducibility. In this work, we present a high-performance lab-on-a-chip sensor exploiting mid-IR spectroscopy for liquid sensing. It is operating at 6.1 μm wavelength and is suitable for robust and flexible real-time *in situ* analysis of the residual water concentration in isopropyl alcohol. This is demonstrated in two experiments. A custom-made 60 μL flow cell is employed to measure only minute amounts of analyte in an inline configuration. In a second approach, the whole sensor is immersed into the analyte to demonstrate sensitive and rapid *in situ* operation on the millisecond time scale. This is confirmed by the ability for time resolved single water-droplet monitoring, while they are mixed into the liquid sample. We obtain a limit of detection between 120 ppm and 150 ppm with a concentration coverage spanning three orders of magnitude from 1.2 × 10^−2^%_vol_ to 25%_vol_ for the flow cell and 1.5 × 10^−2^%_vol_ to 19%_vol_ in the *in situ* configuration, respectively.

## Abstract 2

A sensitive and selective monolithic mid-IR lab-on-a-chip for real-time analysis of water-residuals in solvents with comparable LOD to Karl–Fischer titration.

## Graphical abstract

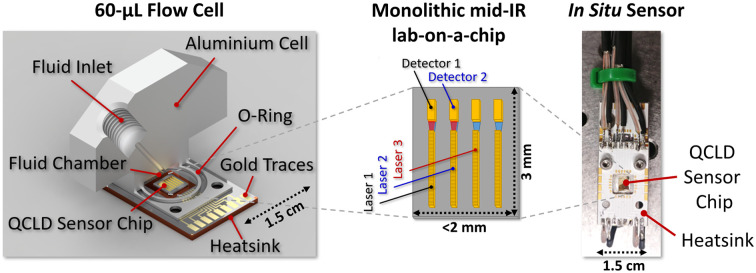

## 1 Introduction

Organic solvents form the foundation of chemical synthesis for pharmaceuticals, agricultural products, polymers, dyes and textiles. The need for pure (water-free) solvents is often a strong requirement for optimum yield and purity of the final products, demanding permanent cleanliness control in terms of residual water concentration. Another field, where monitoring water contamination is of utmost importance, is the petrochemical industry, where identifying water traces in jet-fuels is an important safety measure to prevent ice formation in airplane engines at temperatures below 0 °C.

Current analytical methods for water analysis are strongly dominated by Karl Fischer (KF) titration. It is usable in a wide variety of matrices including organic solvents, oils, petrochemical and pharmaceutical products and jet-fuel.^1–5^ This method is based on the reaction of residual water with sulfur dioxide and iodine in an alcohol solution containing a base. Quantification in KF titration is typically achieved through coulometric or volumetric techniques. While both individually yield consistent results, the compared values differ strongly depending on the hydrocarbon composition.^6^ This typically results in a satisfactory intra-laboratory accuracy, but a poor inter-method and -laboratory performance. Consequently, the results from each KF procedure have to be compared to certified water standards to obtain absolute values. These complications, together with the need for typically toxic and expensive consumables, result in offline analysis only, where the time-demanding sampling and the risk of sample contamination during handling, can outweigh the good sensitivity and selectivity of the KF technique.^5,7,8^ In comparison, alternative (chemical) analysis methods^9^ have advantages for selective criteria like no need for reagents (*e.g.* electrochemical methods,^10^ visual inspection method, drying to constant weight or distillation), the possibility for online monitoring (*e.g.* fluorescent-based techniques^9,11^) or their robustness and ease of use and calibration (*e.g.* electrochemical and -physical methods^9,12^). However, they often suffer from several drawbacks, especially a lack of selectivity and accuracy, making the KF method still the accepted gold standard for water determination in organic solvents.‡‡*E.g.* ASTM standard method D6304 with water evaporator accessory (WAP), considered most comprehensive and accurate available procedure and works for dissolved, free or emulsified water in the concentration range 10 to 25 000 ppm.

Typical values for the limit of detection (LOD) of the KF method are 10 s of μg of detectable water with both (coulometric and volumetric) methods. Depending on reasonable sample portions being used, typically 5–10 grams, this corresponds to LODs for commercial products in the low ppm-range: *e.g.* 1 ppm (10 g sample, coulometric) or 12.5 ppm (5 g sample, volumetric).^7^ They are confirmed by current literature.^8^

In contrast to KF-based analysis and other techniques based on methods from analytical chemistry, traditional (mid-)IR spectroscopy has multiple advantages for residual water monitoring in solvents: since the entire mid-IR spectral range can be covered with each measurement it has a high selectivity. Additionally, the strong fundamental water absorption lines are probed, enabling a high sensitivity. Moreover, in the online geometry, the measurements can be conducted very time efficiently, in the order of seconds. However, also this method has drawbacks – the two most prominent being strong (temperature-dependent) matrix absorption effects and broad absorption features of liquid analytes. The limits for trace detection measurements in state-of-the-art FTIR spectrometers are still often given by the available broadband thermal light sources (*e.g.* globars). They are mostly able to only penetrate up to several micrometers of the analyte for the case of water^13–16^ or slightly more in a low-absorbing buffer like deuterium oxide.^17^ While for moderate analyte concentrations the penetration-depth is less of an issue when analyzing a highly absorbing substance (water) in a low absorbing matrix (solvent), broadband spectrometers are still bulky tools that require a laboratory environment. Consequently, such measurements are often limited to time-consuming offline analysis^13,18^ of significant sample sizes well above microliter volumina,^16^ which can waste precious analytes and prevent inline measurements. Alternatively, inline monitoring can be performed with optical fibers,^19,20^ hollow waveguides,^21^ or by using fluidic cells, together with (functionalized) attenuated total reflection (ATR) crystals.^15,22–24^ While the former often show poor fiber performance and are sensitive to mechanical vibrations, the latter require large setups due to the externally coupled broadband source. Thus, they are all limited in their practical use to certain suitable process environments.^25,26^ While complex or contaminated samples require broad spectral coverage, in many controlled processes it is sufficient to address only a few narrow spectral ranges. They need to contain regions of strong molecule absorption and others with little to no absorption for referencing. This can be achieved employing quantum cascade lasers (QCLs), which have narrow emission linewidths, but are much more powerful than conventional globar sources. Their high intensity leads to significantly increased sample penetration depths of tens of micrometers.^27–31^ This strongly reduces the constraints on the flow cell as well as the complexity of liquid sample preparation and handling.

The QCL is indeed the most prominent laser source in the mid-IR spectral region and recent developments turn the spotlight on QC technology for next-generation sensors. Since the first experimental demonstration of a QCL in 1994,^32^ the devices and technology have achieved various breakthrough developments.^33–35^ Nowadays, QCLs are reliable high-performance mid-IR, light sources operating at room temperature. They have been optimized to address spectroscopic applications by employing various techniques of wavelength selection and control. This includes *e.g.* distributed feedback (DFB) gratings,^36–38^ tunable external-cavity (EC-)QCLs,^33,39^ and broadband frequency combs.^40,41^

Further important milestones towards integrated sensors were the (photovoltaic) detector operation of unbiased QCL structures^42–44^ and the combination of both devices into one single optimized QC active region (AR) for same-wavelength emission and detection (QCLD).^45^ For improved sensor operation surface-sensitive mid-IR plasmonic waveguides can additionally be employed,^46,47^ paving the way for miniaturized, on-chip devices,^46,48^ which suppress beam distortions from standard ridge geometries.^49^ The LOD can be adjusted to the investigated analyte by appropriate design of the waveguide length between the laser and detector. Based on this concept, (low) ppm values have already been realized when analyzing water^46^ or proteins.^48^ Due to the monolithic nature of such sensors, upscaling has minimal impact on the fabrication time or complexity. Another advantage is the much shorter data acquisition time of single wavelength measurements (<1 ms), compared to recording broadband spectra with an FTIR spectrometer (>1 s). This enables real-time monitoring of dynamic processes on millisecond timescales.^48^

**Fig. 1:**
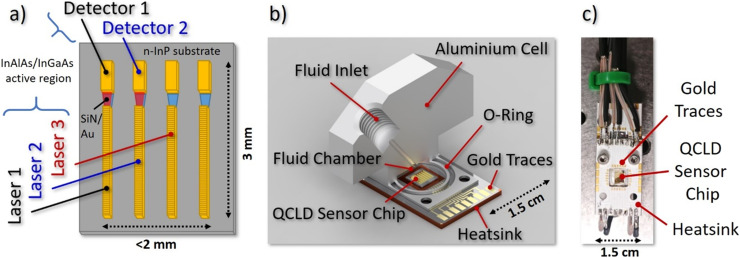
QCLD liquid sensor schemes: a) schematic QCLD sensor array, including 4 pairs of DFB QCLs and QCDs coupled with plasmonic waveguides. The three highlighted devices are used in the experiments. b) Cut through the fluid cell design (volume: 60 μL) attached on top of the QCLD sensor for microliter-scale measurements. The fluid outlet is put symmetrically on the opposite side that is omitted here. c) *In situ* sensor for direct immersion in the liquid. The QCLD sensor array is the shiny gold rectangle in the center of the picture.

In this work we present the robustness and high performance of such a monolithic approach through realizing two compact liquid sensor designs. For this we either implement the QCLD chip (see Fig. 1(a)) into a miniaturized flow cell (see Fig. 1(b)) or use it as an *in situ* probe (see Fig. 1(c)), directly immersing it in the sample beaker. For each of the two designs we perform proof-of-concept absorption spectroscopy experiments with high sensitivity and specificity, measuring the deionized water concentration in the solvent isopropyl alcohol (IPA) as the matrix at ∼6.1 μm wavelength. This demonstrates the various capabilities of the QCLD sensor. They include real-time inline monitoring of well-defined dynamic concentration profiles, a high temporal and spatial resolution in *in situ* measurements, low LODs and high selectivity, no need for (toxic) reagents producing waste (“green monitoring”), the fundamental ability to distinguish between undissolved and dissolved water and a high reproducibility.

## 2 Device design & experimental setup

### 2.1 QCLD device design and characterization

The QCLD device used in the experiments is based on a 37 period InAlAs/InGaAs AR, grown lattice-matched by molecular beam epitaxy on an n-InP substrate. It was fabricated into a ridge-device configuration with 10 μm-wide QCLs facing 15 μm-wide QCDs, separated by a 50 μm-gap. This gap hosts a tapered SiN/Au dielectric-loaded surface plasmon polariton (DLSPP) waveguide for mode confinement with simultaneous sensing capabilities.^48^ To narrow down the spectral emission for addressing individual features of the broad liquid absorption bands, a weakly-coupled DFB grating is implemented into the QCLs. The spectral emission characteristics can be found in the ESI† material Fig. S1. More details on the QCLD-devices and weakly-coupled DFB–QCLs can be found in references.^46,48^

Fig. 1a) depicts a schematic of the 6.1 μm wavelength QCLD sensor chip used as the core of our experiments. It features several pairs of DFB QCLs and QCDs coupled by the DLSPP waveguides. Two pulse generators are used to bias laser 1 and 2 sequentially with 100 ns pulses and a repetition rate of 5 kHz (duty-cycle of 0.05%), with laser 2 being delayed by 260 ns. The delay in biasing is a simple mitigation strategy to avoid electrical crosstalk between neighboring sensor elements, which originates from the compact nature of the on-chip geometry with a shared electrical ground. Their optical emission is detected and converted to a voltage signal by their corresponding (unbiased) QCDs. Optical power and electrical resistance of QC structures are highly temperature-dependent, enabling the use of the neighboring laser 3 as a fast on-chip temperature probe. This allows real-time monitoring of the actual device temperature and to eliminate temperature-induced intensity fluctuations during post-processing of the data. If performed properly, it in principle enables *in situ* operation without external temperature stabilization of the sample or the sensor. The corresponding detector signal and resistance *versus* temperature calibration curves are provided in the ESI† material Fig. S2a. The fourth laser/detector pair displayed in Fig. 1a) acts as a back-up in case of failure of one of the other laser/detector pairs. Thus, it has not been used in the current experiments.

### 2.2 Microliter fluidic cell

The challenge of designing and implementing a miniaturized liquid flow cell lies in the need to combine microliter-scale liquid handling and encapsulation with electrical connectors and a heatsink for the temperature control of the sensor elements. Our prototype flow cell design is depicted in Fig. 1b) and features a solid aluminum body with a filling volume of ∼60 μL. The QCLD sensor chip is mounted on a 1.5 × 2.5 cm^2^ aluminum PCB, providing both: heat sinking as well as electrical connections to the QCLD. The cell design provides a small circular indentation in its center to host the sensor-chip which also functions as the liquid compartment. It is directly attached to the PCB and sealed with a rubber O-ring. The liquid in- and outlet channels are drilled into the topside of the cell and feature a threading for the microtubing connectors, together with 1/16′′ PTFE tubing. Recording a calibration curve is conducted by pumping pure IPA (99.7%) through the cell, while continuously adding small quantities of water in a well-defined manner and letting the system stabilize (see *e.g.* ESI† material Fig. S2b).

### 2.3 Flow cell experimental setup

**Fig. 2:**
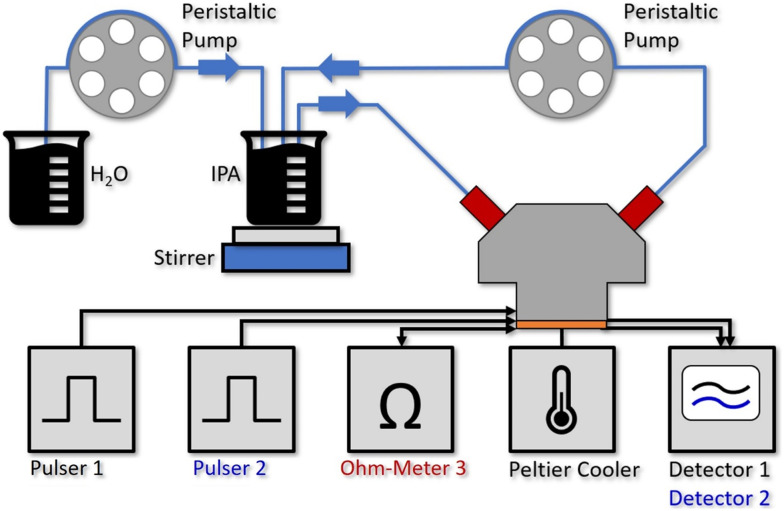
Experimental setup for the water concentration measurements with the 60 μL aluminum flow cell mounted on top of the QCLD sensor, where 2 individual sensors are operated by pulser 1 and 2 sequentially. A third laser is used for on-chip temperature monitoring (Ohm-Meter 3). The Peltier cooler stabilizes the QCLD-sensor to room temperature, whereas the resistance of the third laser changes with fast temperature fluctuations and can be used for compensating them. The detector signals 1 and 2 are read out with a 350 MHz oscilloscope. For establishing defined liquid flows, we use two channels of a multi-channel peristaltic pump. The first one continuously pumps the liquid IPA & water analyte through the fluid cell at a rate of 15 ml min^−1^, while a second channel simultaneously pumps water at a constant rate of 1.5 ml min^−1^ into the IPA beaker, where it is mixed in with a stirrer.

**Fig. 3:**
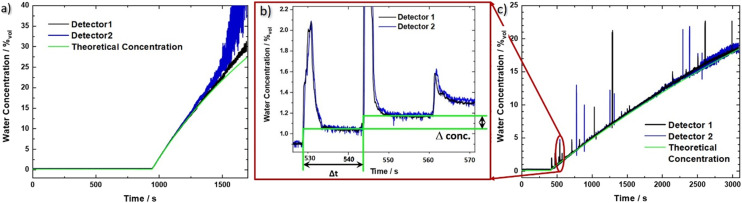
Measured water concentration as a function of time for the QCLD sensor experiments. Both evaluated sensor signals 1 (black) and 2 (blue) are shown together with the theoretical curve (green) for the two different configurations: a) flow cell and b) and c) *in situ* sensor setup. b) Zoom in to the *in situ* sensor signal in the range of 525 to 570 s showing the corresponding signal for three consecutive water droplets. We want to stress the sensitivity and rapid sensor response, demonstrated by its ability to resolve single water droplets on the millisecond time scale (given by the sharp rise time of the water concentration signal). From the time difference Δ*t* and the concentration difference Δconc. we obtain a measured droplet volume of 54 μL between two steps, corresponding well to the theoretical volume of a water droplet between 50 and 60 μL. The calculated pump rate is 216 μL min^−1^ for two consecutive droplets compared to the set value of 200 μL min^−1^ (pump resolution: ∼1%).

Fig. 2 shows the setup for the flow cell experiment. Laser 1 and 2 are driven by pulser 1 and 2, while the resistance of laser 3 is continuously monitored. The detector signals are recorded on a 350 MHz oscilloscope (Teledyne LeCroy HDO4034 2.5 GSPS). The sensor PCB is mounted on a Peltier cooler, stabilized to 20 °C. Due to the low amount of power being dissipated (≪20 mW at the duty-cycle of 0.05%) for driving the individual sensor elements together with the use of a Peltier cooler and the described temperature correction based on laser 3, we do not expect a relevant heating of the liquid even for the 60 μL flow cell. In fact, this can also be seen by the good overlap between the two experimental water concentration curves with the theoretical one in Fig. 3a).

### 2.4 Sampling and data acquisition

The flow cell is filled with IPA (purity: 99.7%) and stabilized to 20 °C when starting the experiment. After the system thermalized for about 20–30 s, pure IPA is pumped from an external beaker through the fluid cell in a closed cycle loop at a constant rate of 15 ml min^−1^ (see Fig. 2). The small temperature increase to 22 °C, caused by the liquid at ambient temperature being pumped through the cell results in a slight drop of the measurement signal and resistance of the on-chip temperature probe (see ESI† material Fig. S3a)).

After running the pump for 15 min to validate a stable signal, the second channel of the peristaltic pump is activated. It continuously adds deionized water to the beaker initially filled with 50 ml of IPA at a rate of 1.5 ml min^−1^ which is immediately mixed into the solution using a stirring bar. Since the pumping rate through the flow cell is 10 times the rate of adding the water, we can assume a similar water concentration in the whole closed loop cycle. Due to the continuously increasing water concentration in the analyte beaker, the detector signals drop exponentially over time. The raw data for the measured detector signal and laser resistance of the on-chip probe are shown in the ESI† material Fig. S3a.

## 3 Experimental results & discussion

### 3.1 Flow cell measurements

Using the prior recorded temperature and detector signal calibrations (ESI† material Fig. S2a) and b)), the water concentration in the analyte beaker can be directly obtained as a function of time. The signals for the two sensors are shown in Fig. 3a) (black and blue line, respectively), together with the theoretical water concentration curve (green line). It has been calculated from the water flow rate of the calibrated peristaltic pump into the IPA beaker, and the initial IPA volume. In the beginning the raw data jumps due to the warmer IPA, which is being pumped through the flow cell (see ESI† material Fig. S3a). However, after applying the temperature compensation method, this jump vanishes, indicating its proper functioning. For concentrations of up to 25%_vol_ the experimental data can be very well described with the theoretical concentration curve. At higher concentrations, however, pronounced deviations indicate the limit of the traditional Beer–Lambert law, which considers absorption effects but not the substantial change in refractive index at higher densities of the sample. Additionally, detector 2 shows an increasingly reduced signal-to-noise ratio, originating from a lower initial signal level, which is further significantly reduced due to the exponential absorption process (Beer–Lambert law). The worse noise performance of sensor 2 as compared to sensor 1, most likely originates from fluctuations in the fabrication process, leading to local defects and a reduced device performance.

The LOD in our measurements is defined as three times the standard deviation of the concentration signal for pure IPA over the calibration line. We obtain a LOD value of 1.2 × 10^−2^%_vol_, corresponding to 120 ppm (averaging about 170 samples, leading to a data rate of 1 sample/s). This translates to a concentration range of operation of our sensor between 1.2 × 10^−2^%_vol_ and 25%_vol_, or 120 ppm to 250 000 ppm. The sensor covers most of the relevant range where KF titration is being used but can in principle be tailored to fit any similar application by adapting the distance between laser and detector. Its significant advantage is a much lower amount of liquid that is probed. In particular, that amounts to 60 μL, *i.e.* 47 mg, of IPA for the QCLD sensor as compared to between 10 g and 5 g of sample with KF titration for a LOD of 1 ppm (coulometric) and 12.5 ppm (volumetric), respectively.^7^ This enables the use of the QCLD sensor in real time, inline monitoring, including bypass liquid stream flows. When comparing similar amounts of probed sample volumes, the LOD of KF titration scales, given by the smallest theoretical increment of current (coulometric method) and titrant (volumetric method) that can be achieved^7^ linearly to 200 ppm and 1250 ppm for 50 mg of analyte. This is significantly higher than with our QCLD.

### 3.2 *In situ* sensor experiment

**Fig. 4:**
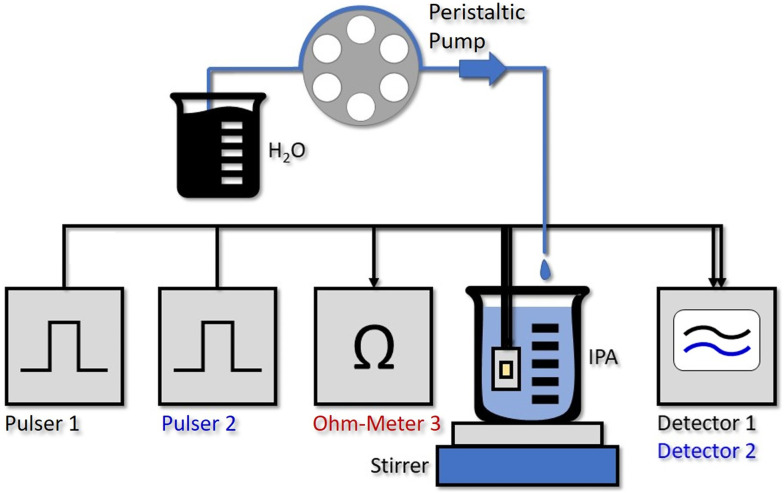
Experimental setup of the water concentration measurement with the *in situ* QCLD sensor. Pulser 1 and 2 drive the two measurement lasers sequentially. While no active temperature control of the QCLD is implemented and it is thermalized through the direct contact with the liquid, the resistance measurement of Ohm-Meter 3 is again used for improved temperature-fluctuation compensation. The detector signals 1 and 2 are read out with a 350 MHz oscilloscope. For establishing a continuous increase of the water concentration in the IPA beaker we use one channel of a multi-channel peristaltic pump. It adds single droplets of water at a slow rate of 0.2 ml min^−1^, which are subsequently rapidly mixed into the solution using a stirring magnet.

By stripping away the flow cell, we can design a simplified system, capable of *in situ* sensing. Our immersion probe is depicted in Fig. 1c) and consists of the QCLD sensor chip mounted on and wire-bonded to the same PCB as before. This is mainly done for mechanical stability and stable electrical contacts, but in principle the sensor could be realized in an even much smaller fashion. It is directly immersed and operated in the analyte beaker without any additional protective measures for the QCLD-chip and its electronic and optical components (experimental setup see Fig. 4). This shows the robustness and versatility of our monolithic sensor approach, while still maintaining high-performance operation. Such a configuration has two main advantages: first, same as for the flow cell no sampling of the analyte is required and second, the sensing is performed *in situ* and in real time, which is the most rapid way to monitor changes of the analyte composition. We performed a similar experiment as for the fluidic cell with two lasers of the QCLD sensor being pulsed sequentially, while their detector signals are read out with the oscilloscope. Simultaneously the temperature, *i.e.* resistance, of the third laser is monitored. The whole sensor chip is immersed in an IPA-filled beaker with a stirrer. It is remarkable, that no active temperature stabilization of the chip itself is being used. The whole thermalization of the QCLD sensor happens through the direct contact with the liquid and continuous operation. The measurement procedure follows a similar protocol as before: data acquisition is started first, followed by the activation of the stirrer a few seconds later. This causes a temperature drop at the sensor, which increases the intensity and therefore the detector signal (see ESI† material Fig. S3b). After about 8 minutes for validating the stable operation of the sensor, the peristaltic pump is activated, slowly dripping water into the IPA beaker at a steady flow rate of 0.2 ml min^−1^. The slow flow rate allows us to investigate the long-term stability of the sensor under regular operation conditions. Due to the geometry of the setup, the stirrer causes the added water drops to swirl over the sensor before completely mixing with the rest of the liquid. The temperature and detector signal calibration curves are applied to calculate the measured water concentration as a function of time. They are plotted in black and blue for sensor 1 and 2, respectively, together with the theoretical curve (green) in Fig. 3c). A good agreement with the predicted concentration can be observed up to 19% of water concentration. Similar to the flow cell experiment, the temperature influence is canceled by applying a post-processing routine with the data of the on-chip probe. This can be observed from the initially constant signal in pure IPA and the monotonic water concentration increase when disregarding the “spikes” in the signal shown in Fig. 3b) and c). These spikes are the most peculiar difference to the previous flow cell experiment. They correspond to single water droplets that hit the liquid in the beaker and are subsequently being swirled over the sensor itself (hence the sharp increase of water concentration signal), before being completely mixed into the solution. The height of the spikes varies for different water droplets, even though the stirring of the solution is nominally kept constant (*i.e.* constant rotation of the stirring magnet). Still, we expect that the mixing conditions and therefore the mixing efficiency slightly vary for subsequent water droplets, resulting in the observed variation in spike height. The ability to resolve impinging, individual water droplets demonstrates the fundamental ability of the sensor to distinguish between dissolved and undissolved water in the solvent. The spike rise time is direct evidence of the high sensitivity and rapid response time of our sensor down to the millisecond time scale. It is only limited by the pulser frequency and the averaging of multiple pulses. It is further possible to calculate the time constant of the mixing process, as well as the volume of the added water drops. As shown in Fig. 3b), we can extract a droplet period of 15 s from the subsequent spike positions, while the following plateau after each spike can be used to calculate the volume of each droplet through the indicated concentration change Δ_conc_. For example, the first displayed droplet has a volume of 54 μL, which agrees very well when compared to the theoretical value of about 50–60 μL. In addition, we obtain a flow rate directly calculated from two consecutive droplets of 216 μL min^−1^, also corresponding very well to the set value of 200 μL min^−1^ (pump resolution: ∼1%).

As can be seen from Fig. 3c), the measured signal follows the theoretical curve until around 19%, where it starts to become increasingly noisy due to the higher water concentration. The LOD measured with IPA is 1.5 × 10^−2^%_vol_, 150 ppm, for an averaging that corresponds to a sampling rate of 1 sample/s. The setup with the immersion sensor allows to cover the concentration range from 150 ppm to 190 000 ppm. This is remarkable, especially when considering that the QCLD sensor is not actively temperature stabilized but simply thermalized in the liquid solution.

### 3.3 Absorbance calculation

Following the experimental results, we also analyzed the calibration lines for both sensors through calculating the absorbance as a function of water concentration, to demonstrate that the sensor operates according to the Beer–Lambert law. This law can be used when fulfilling certain criteria such as collimated and monochromatic light beams, homogeneously distributed analytes, low (enough) substance concentrations and negligible scattering in the analyte.^50^ The absorbance *A* can then be calculated from the measured detector signals:
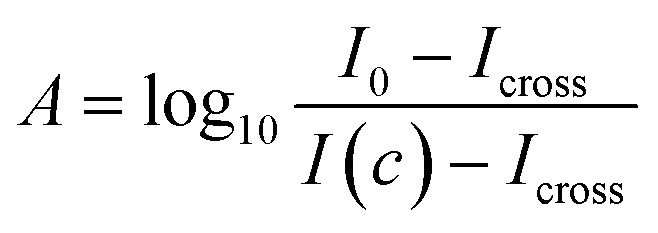
with the reference signal of the pure IPA matrix *I*_0_, the water-concentration dependent detector signal *I*(*c*) and the electrical crosstalk signal *I*_cross_ measured in pure water when there is no remaining optical signal on the detector because of the strong water absorption.

**Fig. 5:**
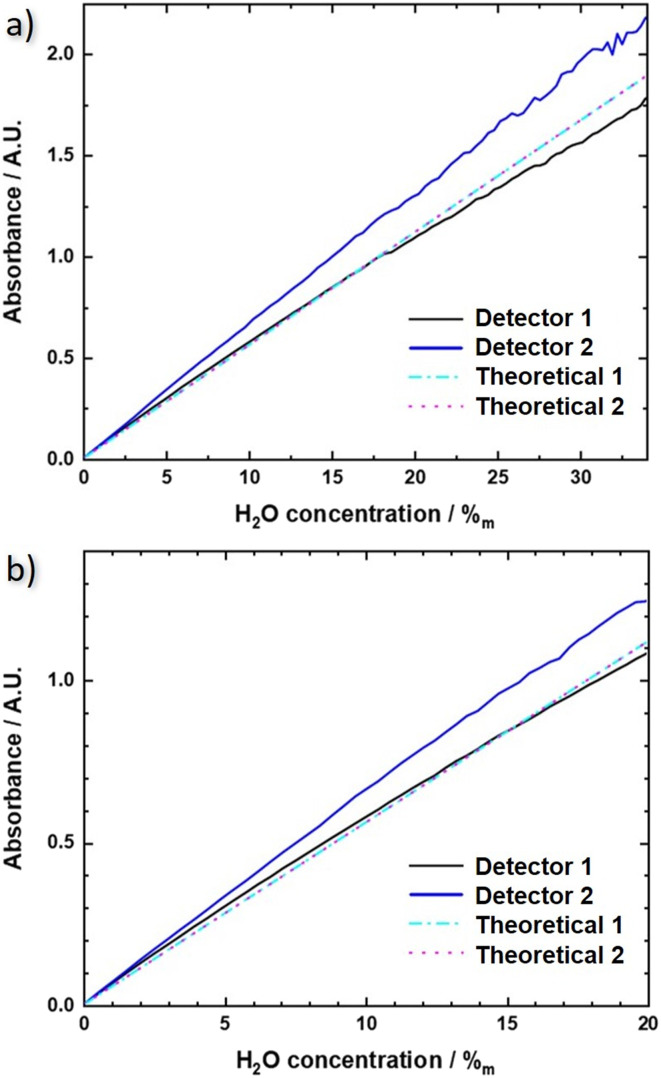
QCLD sensor calibrations line measurements for both types of sensor configurations: a) flow cell and b) *in situ* sensor. The theoretical absorbance is calculated with the absorption spectra of water and IPA for the laser emission spectra 1 and 2 (cyan, magenta) and compared to the experimental absorbance, calculated from the acquired data of the two sensors (black, blue).

The resulting experimental QCLD sensor reference lines are shown in Fig. 5a) for the flow cell and b) for the *in situ* sensor and both lasers (black and blue lines, respectively). For comparison, theoretical absorbance curves are plotted in the same figures (cyan and magenta), calculated for an actual interaction section of 48 μm using the normalized laser spectra and the absorbance spectra shown in the ESI† material Fig. S1b). The calculation was performed for the whole range of water concentrations displayed in Fig. 5a) and b), respectively. Sensor 1 shows very good agreement between theory and experiment, especially for the *in situ* configuration. The mainly relevant deviations are observed for the fluidic cell at water concentrations above 27% where the approximation of the Beer–Lambert law is not valid any more. The situation is different for sensor 2. It consistently shows a lower absorbance in the theoretical curve, which might originate from the applied measurement routine. Since the QCLDs are pulsed sequentially with sensor 2 being delayed by 260 ns only, with respect to sensor 1, additional heating effects may occur, which are not considered in the calculations so far. Additionally, but somewhat less relevant in this case, a temperature increase causes a red-shift in the spectral emission in the direction of the strong water absorption. We probably therefore calculate lower absorbance values than experimentally measured.

In a quantitative comparison, we can extract the actual slopes of the different QCLD sensor calibration lines and obtain for the flow cell sensor in Fig. 5a): 50.9 mAU/%_m_ (H_2_O) and 64.1 mAU/%_m_ (H_2_O) for detector 1 and 2, respectively, and 55.7 mAU/%_m_ (H_2_O) for the theoretical calculation (theoretical 1 and 2). For the *in situ* sensor in Fig. 5b) we get 53.8 mAU/%_m_ (H_2_O) and 63.2 mAU/%_m_ (H_2_O) for detector 1 and 2, respectively, and 56 mAU/%_m_ (H_2_O) for the theoretical calculation (theoretical 1 and 2). This again demonstrates the limitations of the approximations used in the traditional Beer–Lambert law to theoretically calculate the absorbance of a higher density liquid sample.

In addition, it is interesting to see that the QCLD sensor works for water concentrations up to above 2 A.U., which is an absorbance range that is typically difficult to be accessed with conventional FTIR spectrometers.^51^

In future work the liquid handling and encapsulation of the fluidic analyte can be further optimized by implementing microchannels directly bonded to the plasmonic waveguide, allowing to easily load and handle the fluidic analyte.^52,53^ Additional on-chip micromixers can be connected to the microchannels^53^ ensuring homogenous analyte distribution while avoiding mechanical vibrations *e.g.* from using a stirring magnet. Such an improved analyte manipulation will help to further expand the application scenarios of the QC-based sensor to *e.g.* environmental monitoring and clinical diagnosis. An optofluidic microcavity implemented to a QCL was already demonstrated in the past, based on a combined SU-8 and PDMS chamber,^54^ as it is typically used for devices in the visible/near-IR spectral range.^52,53^ While very low microliter-scale volumes could be realized this way, the involved polymers have high absorption losses throughout the mid-IR spectral range. Consequently, remaining polymer residuals from imperfections in device fabrication or polymer-cells in the optical path, will result in significantly increased modal losses. Therefore, the current development of a spin-coating process for micrometer-scale polyethylene-based thick films will help to boost future improved mid-IR chip-scale optofluidic microcavities.^55^ Polyethylene is an excellent candidate for mid-IR applications due to its highly suitable refractive index profile, including broadband low losses throughout the entire mid-IR spectral range.^55^

## 4 Conclusion

In conclusion, we showed two sensor concepts for the analysis of residual water concentration in IPA, employing a QCLD sensor working at 6.1 μm wavelength. One approach is based on a 60 μL fluidic cell for inline measurements and the second is performed in an *in situ* configuration for direct immersion in the analyte. In two proof-of-concept experiments the water concentration in IPA was measured between ∼120/150 ppm and 250 000/190 000 ppm, which includes the ability to measure high absorbance values of water above 2 A.U. The current results show the sensitive and selective operation and therefore high potential of the monolithic sensor concept for rapid and dynamic liquid spectroscopy measurements in the mid-IR spectral range. This includes single water droplet resolution on the microsecond time scale. By further exploiting suitable measurement and averaging techniques as well as additional post processing schemes, the LOD value can be further reduced by one order of magnitude.^46^ In order to push the LOD to even lower values, a more efficient laser design can be developed. This will on one hand enable continuous-wave operation of the sensor improving data averaging and time resolution. On the other hand, more powerful lasers are advantageous, since they can penetrate through thicker liquid sample layers. Applying strongly coupled DFB gratings that enable single mode emission is another important step towards more stable laser operation and therefore a more robust high-performance mid-IR sensor. This paves the way for future experiments in many other liquids.

## Author contributions

F. P., B. B. and B. H. designed the liquid experiments; F. P. performed the liquid measurements and characterized the quantum cascade devices; B. S. and D. R. designed and fabricated the quantum cascade devices; H. D. and A. M. A. grew the quantum cascade structures; F. P. analysed the results; G. S. and B. H. supervised the research activity; F. P. and B. H. wrote the manuscript with editorial input from B. B., B. L. and G. S.; all authors read the manuscript and commented on the paper.

## Conflicts of interest

The authors declare no competing interests.

## Supplementary Material

LC-023-D2LC00724J-s001
