# Supplementary material for: Beyond Karl Fischer titration: a monolithic quantum cascade sensor for monitoring residual water concentration in solvents
Source: Lab Chip. 2023 Feb 13;23(7):1816–24. doi: 10.1039/d2lc00724j (PMC10045895; doi:10.1039/d2lc00724j)
Supplement: LC-023-D2LC00724J-s001 [file LC-023-D2LC00724J-s001.pdf]

Electronic Supplementary Material (ESI) for Lab on a Chip.
This journal is © The Royal Society of Chemistry 2023

Journal Name

ARTICLE TYPE

Cite this: DOI: 00.0000/xxxxxxxxxx

# SUPPLEMENTAL MATERIAL: Beyond Karl Fischer titration: a monolithic Quantum Cascade sensor for monitoring residual water concentration in solvents †

Florian Pilat,*[a] Benedikt Schwarz,[a] Bettina Baumgartner,[b] Hermann Detz,[a,c] Aaron M. Andrews,[a] Bernhard Lendl,[b] Gottfried Strasser,[a] and Borislav Hinkov*[a]

Received Date
Accepted Date

DOI: 00.0000/xxxxxxxxxx

Figure S1. shows the spectral emission characteristics of laser 1 (black) and laser 2 (blue) which are part of the used QCLD sensor chip. It is superimposed with the absorbance curves of $H_2O$ (cyan)* and IPA (magenta)† for an interaction length of 48 $\mu$m. In Fig. S1.a) the entire mid-IR spectral range between 2.5 $\mu$m and above 20 $\mu$m is depicted, while Fig. S1.b) displays only the relevant part where the laser emission is present. The effect of the weakly coupled DFB gratings can be seen as still multiple modes are present in the laser emission spectra. Figure S2. shows the QCLD calibration curves for: a) temperature dependence of the detector signals of sensor 1 (black) and sensor 2 (blue) and of the resistance of laser 3 in pure IPA. Figure S2.b) detector signal of sensor 1 (black) and sensor 2 (blue) as function of the water concentration. The later is performed by pumping pure IPA (99.7 %) through the cell while continuously adding small quantities of water.

Figure S3.a) shows the raw data of the experiment performed in the fluidic cell, as presented in the main article. It shows the time-dependent signals of sensor 1 (black) and sensor 2 (blue) as well as of the resistance of laser 3 (red) which acts as a fast on-chip temperature probe. In the beginning pure IPA is resting in the cell, then the peristaltic pump is started, causing the warmer IPA from the beaker to flow through the cell. This increases the temperature inside the cell from 20 °C (setpoint of the Peltier cooler) to 22 °C (ambient temperature of the IPA beaker), leading to an initial small drop in the signals. When continuously pumping the IPA for 15 min, the signal remains stable until the pumping of the water into the analyte beaker is started. The following signal decay at both detectors 1 (black) and 2 (blue) is caused by the gradually increasing absorption due to the increasing water concentration.

Figure S3.b) displays the equivalent raw data time traces for the *in situ* experiment when immersing the QCLD sensor in the liquid analyte. The sharp small signal rise after a few seconds of detector 1 (black) and detecor 2 (blue) results from switching on the stirring in the beaker, which in turn has a cooling effect on the sensor and thus both detector signals slightly increase.

[a] *Institute of Solid State Electronics and Center for Micro- and Nanostructures, TU Wien, 1040 Vienna, Austria.*
*E-mail: *florian.pilat@tuwien.ac.at; *borislav.hinkov@tuwien.ac.at*
[b] *Institute of Chemical Technologies and Analytics, TU Wien, 1060 Vienna, Austria.*
[c] *Central European Institute of Technology (CEITEC), Brno University of Technology, 621 00 Brno, Czech Republic.*

* Y. Maréchal, "The molecular structure of liquid water delivered by spectroscopy in the whole IR region completed with thermodynamics data", *Journal of Molecular Structure* **1004**(1-3), 2011. doi: 10.1016/j.molstruc.2011.07.054.

† T. L. Myers, R. G. Tonkyn, A. M. Oeck, T. O. Danby, J. S. Loring, M. S. Taubmann, S. W. Sharpe, J. C. Birnbaum, and T. J. Johnson, "IARPA / PNNL Liquid Phase IR Spectra", in *NIST Chemistry WebBook, NIST Standard Reference Database Number* **69**, Eds. P.J. Linstrom, W.G. Mallard, National Institute of Standards, and Technology, Gaithersburg MD, 20899, 2018. doi: 10.18434/T4D303.

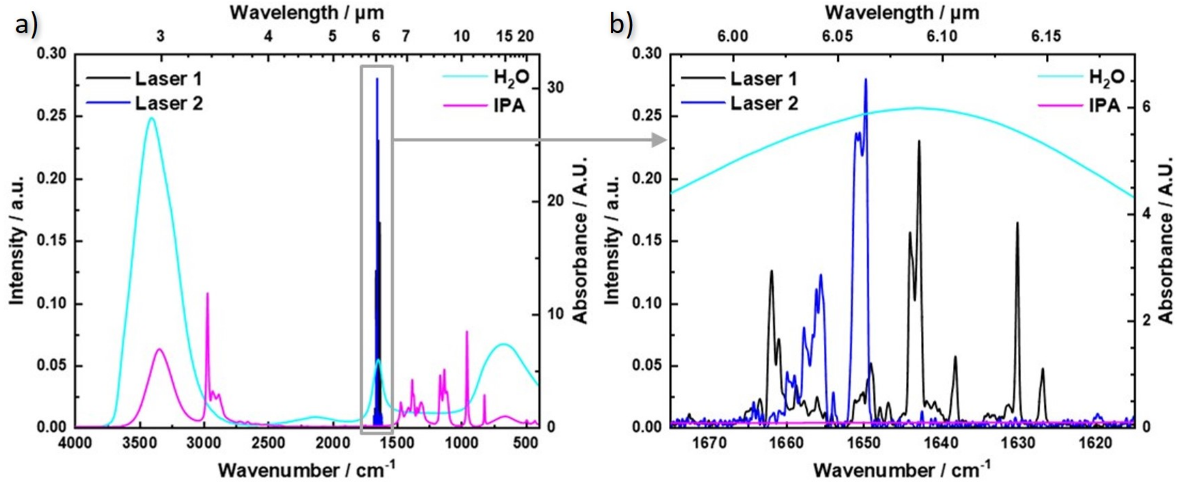


Fig. S1. Emission spectra of the QCLD sensor lasers 1 (black) and 2 (blue) working sequentially and absorbance spectra of $H_2O$ (cyan)* and IPA (magenta)† for a 48 µm interaction length. a) Overview of the entire mid-IR spectral range from 2.5 $\mu$m to above 20 $\mu$m and b) zoom-in on the laser modes that are used for the sensing experiments.

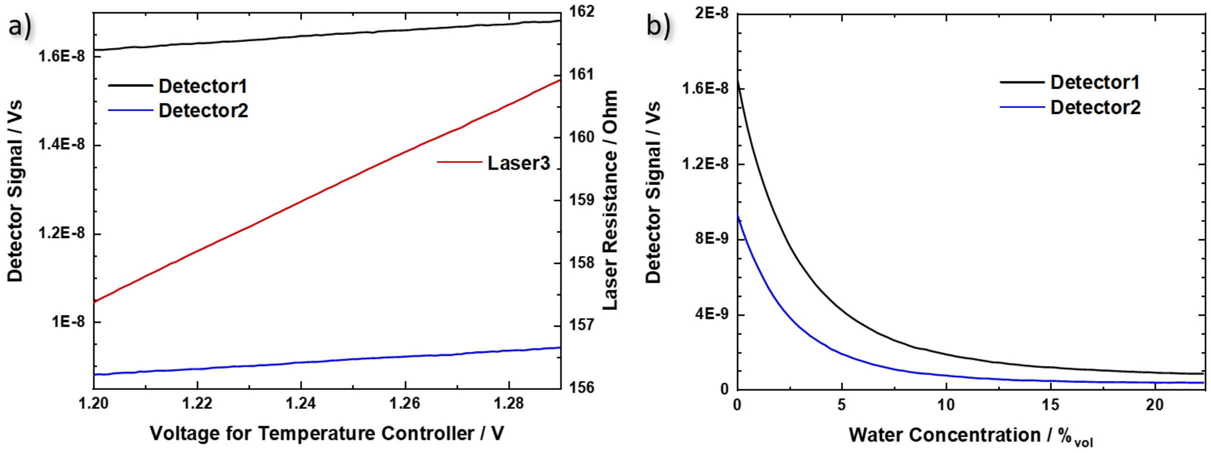


Fig. S2. Calibration curves of the QCLD sensor chip: a) temperature dependence of the detected signals of sensor 1 (black) and 2 (blue) and of the resistance of laser 3 (red) in pure IPA, b) detector signals of sensor 1 (black) and 2 (blue) depending on the water volume concentration in IPA.

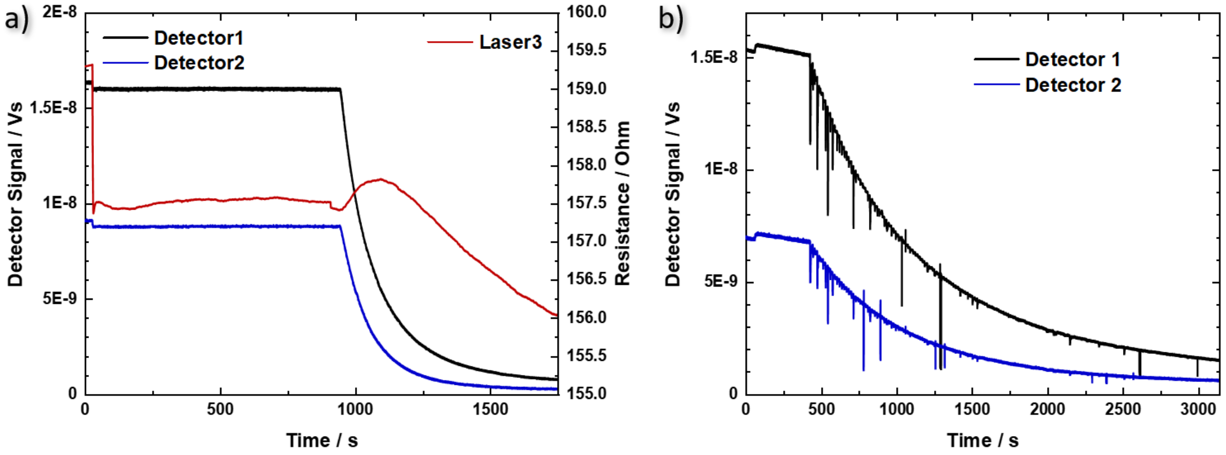


Fig. S3. Raw time traces of the QCLD liquid sensor experiments, sensor signals 1 (black) and 2 (blue) and resistance of laser 3 (red): a) flow cell setup, b) *in situ* sensor setup.
